# The use of live food as a vehicle of soybean meal for nutritional programming of largemouth bass *Micropterus salmoides*

**DOI:** 10.1038/s41598-021-89803-2

**Published:** 2021-05-25

**Authors:** Giovanni S. Molinari, Michal Wojno, Karolina Kwasek

**Affiliations:** 1 Center for Fisheries, Aquaculture, and Aquatic Sciences, Southern Illinois University, Carbondale, IL USA; 2 Center for Fisheries, Aquaculture, and Aquatic Sciences, Southern Illinois University, 1125 Lincoln Dr. Life Science II, Room 175, Carbondale, IL 62901 USA

**Keywords:** Ichthyology, Epigenetics, Cytokines, Tumour-necrosis factors

## Abstract

Nutritional Programming (NP) has been studied as a means of improving dietary plant protein (PP) utilization in different fish species. This study investigated the use of enriched live feed as a vehicle for NP in larval fish. The objective of this study was to determine the effect of NP induced during the larval stage via PP-enriched live feed on: (1) growth performance; (2) expression of genes associated with inflammation and any morphological changes in the intestine; and (3) muscle free amino acid composition in largemouth bass (*Micropterus salmoides)* during its later life stages. Two diets were used in this study, a fish meal (FM)-based diet, and a soybean mean (SBM)-based diet, serving as the PP diet. There were 4 groups in this study. The two control groups, ( +) Control and (−) Control, were not programmed and received the FM-diet and SBM-diet, respectively throughout the whole trial after the live feed stage (27–122 days post hatch (dph). The next group, programmed, was programmed with SBM-enriched *Artemia* nauplii during the live feed stage (4–26 dph) and challenged with the SBM-diet during the final stage of the study (79–122 dph). The final group, non-programmed, did not receive any programming and, was challenged with the SBM-diet during the final stage of the study. The programmed group experienced a significantly higher (%) weight gain during the PP-Challenge than the non-programmed group. In addition, the live feed programming resulted in significantly longer distal villi, and a higher villi length to width ratio, compared to the non-programmed group. No significant effects on free amino acid composition and gene expression were observed between the programmed and non-programmed group, except for an increased post-prandial concentration of free proline in the programmed group. The results of this study support use of live feed as a vehicle for nutritional programming and improving the growth performance of largemouth bass fed with a SBM-based diet.

## Introduction

As the aquaculture industry grows, there is building pressure to find more suitable protein sources to fully replace fishmeal (FM) and other high-quality plant protein concentrates in fish diets. Different types of plant proteins (PP) have been studied extensively as additives in diets to reduce the use of FM in aquaculture^1–5^. Currently the most commonly used FM replacement is soybean meal (SBM). Its inclusion level, however, is limited. The induction of intestinal inflammation from SBM has led to significantly decreased growth performances when fed at levels exceeding 25%^6–10^. For example, in a study done on a carnivorous fish species Rainbow Trout (*Oncorhynchus mykiss*), the replacement of FM with SBM resulted in significantly lower weight gain and an increased FCR during the study^11^. Miao et al.^12^ also found that SBM levels over 50% in the diets of northern snakehead (*Channa argus*) significantly decreased weight gain and specific growth rate as soon as 21 days of feeding with the SBM diet.

One approach to mitigating the negative effects of dietary PP and improving the growth performance of fish fed PP-based diets is nutritional programming (NP). NP is a concept that an organism can be ‘programmed’ to utilize a certain dietary component through exposure during early life stages. Traditionally, NP has been induced using dry feed during the juvenile stage of the fish’s development. Previous studies have achieved positive results using this form of NP^3,13–15^. However, the digestive tract of the fish is most responsive to different environmental factors, including diet, during the larval stages, immediately after hatching. And therefore, it seems most effective to induce NP during that stage, when the digestive tract experiences a significant amount of phenotypic plasticity during its development^16^.

A significant limitation to larval NP however, is poor food intake and the inefficiency of dry feed utilization due to poor digestive tract development at such a young age. According to Sales^17^, larval fish are 2.5 times more likely to experience mortality when fed with dry diets as opposed to live feeds. Additionally, studies have found that replacing live feed with dry diets at first feeding negatively affects the growth of larval fish^18–21^. Larval goldfish (*Carassius auratus*) showed significantly reduced growth and survival on dry feeds, compared to fish fed with live food and the reduction in growth was associated with weak tryptic activity in the intestinal tracts^18^. In addition to reduced digestive activity, a reduction in feed intake has been observed in live feed replacement studies. Adekunle and Joyce^19^, reported that hybrid catfish (*Herterobranchus bidorsalis x Heterobranchus longifilis*) had presented lower feed intake of the dry diets compared to *Artemia* nauplii, and this led to reduced growth and survival of the newly-hatched catfish.

In order to avoid the negative effects of dry diets at first feeding, some researchers have utilized enriched live feed as a vehicle for nutritional components in larval fish. Past studies have found that *Artemia* enrichment is an effective vehicle for probiotics^22^, iodine^23^, and highly unsaturated fatty acids^24^. Based on these findings, we tested the use of *Artemia* nauplii as a vehicle for SBM to induce NP. The *Artemia* are filter feeders and absorb their nutrients from the water^25^. We hypothesized that by enriching the *Artemia* with SBM solution, we could program the larval largemouth bass (LMB) (*Micropterus salmoides)* at first feeding, while still keeping the natural feeding regime of this fish to ensure nutrient delivery and high feed intake. The objectives of this study were to determine the effect of NP with dietary PP induced during the first feeding of larval LMB using live food as a vehicle on: (1) LMB growth performance; (2) Expression of genes associated with inflammation and any morphological changes in the intestine; and (3) Muscle free amino acid (FAA) composition in LMB during its later life stages used as an indicator of dietary amino acid availability.

## Materials and methods

The feeding trial was conducted in the Center for Fisheries, Aquaculture, and Aquatic Sciences at Southern Illinois University-Carbondale (SIUC), IL. All experiments were carried out in strict accordance with the recommendations in the Guide for the Care and Use of Laboratory Animals of SIUC. The SIUC Institutional Animal Care and Use approved all of the protocols performed (protocol# 18-051). All researchers were trained in accordance with SIUC Institutional Animal Care and Use requirements. This study was carried out in compliance with the ARRIVE (Animal Research: Reporting of In Vivo Experiments) guidelines. During fish handling, anesthesia was performed using water bath immersion in tricaine methanesulfonate (MS222) at a recommended concentration (25 mg/l), and all efforts were made to minimize pain, stress, and discomfort in the animals. Humane endpoints were used in this study, fish were euthanized within 24 h if they showed significant signs of deteriorating quality of life. Fish were observed each day for signs of distress. No fish in this study had to be euthanized prior to the completion of the experiment. The ethical guidelines of handling and fish care follow the same protocols outlined in a previous study conducted at the same facility^26^.

The experiment was carried out using a semi-recirculated aquaculture system with two mechanical (sand) filters (Pentair, Minneapolis, MN) and two bio-filters. The water in the system was exchanged at a daily rate of 20% during the larval stage (4–26 dph) and 50% for the rest of the study. The system consisted of 40 (100 L) light blue tanks. The average water temperature was 23.8 °C (± 1.3), the pH was 7.70 (± 0.29), and the salinity was kept between 1 and 3 ppt during the live feeding stage to prolong the viability of the live food^27^. The photoperiod consisted of 14 h of darkness and 10 h of light, with the overhead lights on 8:00–18:00.

### Feed preparation and formulation

Prior to mixing, the dry components of the diet were ground to a fine particle size (~ 0.5 mm) using a centrifugal mill (Retsch 2 M 100, Haan, Germany). Once the dry components were ground, all ingredients in the diet were mixed (HCM450 Vertical Cutter Mixer, Hobart, Troy, OH) to achieve uniform dispersion. To produce small pellet sizes, half of the mixture was extruded (Caleva Extruder 20, Sturminster Newton Dorset, England) and spheronized (Caleva Multibowl Spheronizer, Sturminster Newton Dorset, England). The other half of the mixed diets were run through a food chopper (General Slicing SD-50, General Inc., Weston, FL), to obtain a variety of larger pellet sizes. The pellets were dried (Harvest Saver Tray Dryer, Commercial Dehydration Systems Inc., Eugene, OR) to remove moisture from the diets. After drying, all pellets were separated by size using a vibratory sieve shaker (Retsch AS 200 Basic, Haan, Germany).

The diets were formulated to be isolipidic and isonitrogenous, with crude protein and lipid levels of 48.9% and 10%, respectively. The SBM diet had a full fishmeal replacement with soybean meal (46.3%) and soy protein isolate (15.4%). Soy protein isolate was utilized to adjust dietary crude protein level while also leaving room for other ingredients in the formulation, including a minimum level of starch to allow expansion of the diets. The formulations for each diet are listed in Table 1. The amino acid compositions of the diets are laid out in Table 2. In this study, the SBM diet serves at the PP-based diet.

**Table 1:** Feed formulation of experimental diets.

Ingredients (g/100 g)	FM	SBM
Fishmeal^a^	63.8	–
Soybean Meal^b^	–	46.3
SPC^c^	–	15.4
Krill meal^d^	10.0	10.0
CPSP^e^	5.8	5.7
Dextrin	5.3	–
Fish oil^f^	3.9	7.1
Soy lecithin^g^	4.7	4.7
Mineral mix^h^	2.4	2.4
CaHPO_4_	–	1.4
Vitamin mix^i^	2.0	2.0
Vitamin C^j^	0.1	0.1
Choline chloride	0.1	0.1
Methionine	–	0.5
Lysine	–	2.3
Threonine	–	0.1
Taurine	0.9	0.9
Guar gum	1.0	1.0
**Proximate composition (%)**		
Lipids	17.25 (± 0.47)	16.89 (± 0.08)
Crude protein *(N* × 6.25*)*	54.51 (± 0.57)	53.30 (± 0.13)
Ash	15.39 (± 0.09)	9.10 (± 0.27)
Energy (kcal/g)	5.23 (± 0.03)	5.45 ± 0.04)

*FM* fishmeal, *SBM* soybean meal, *SPC* soy protein concentrate, *CPSP *fish protein hydrolysate.

^a^Mechanically extracted menhaden meal, stabilized with 0.06% ethoxyquin (Omega Protein, Reedville, VA, USA).

^b^Solvent extracted soybean meal (Premium Feeds, Perryville, MO, USA).

^c^Crude protein concentration min. 92% (Dyets Inc, Bethlehem, PA, USA).

^d^Proccesed *Euphausia superba* (Florida Aqua Farms, Dade City, FL, USA).

^e^Soluble fish protein hydrolysate (Sopropeche S.A., Boulogne Sur Mer, France).

^f^Cod liver oil (MP Biomedicals, Solon, OH, USA).

^g^Yelkin TS lecithin (Ingredi Co., Baltimore, MD, USA).

^h^Bernhart-Tomarelli mineral mix with 5 ppm selenium in a form of sodium selenite (Dyets, Bethlehem, PA, USA).

^i^Custom Vitamin Mixture (mg/kg diet) Thiamin HCl, 4.56; Riboflavin, 4.80; Pyridoxine HCl, 6.86; Niacin, 10.90; D-Calcium Pantothenate, 50.56; Folic Acid, 1.26; D-Biotin, 0.16; Vitamin B12 (0.1%), 20.00; Vitamin A Palmitate (500,000 IU/g), 9.66; Vitamin D3 (400,000 IU/g), 8.26; Vitamin E Acetate (500 IU/g), 132.00; Menadione Sodium Bisulfite, 2.36; Inositol, 500 (Dyets, Bethlehem, PA, USA).

^j^l-Ascorbyl-2-polyphosphate (Argent Aquaculture, Redmond, WA, USA).

**Table 2:** Dietary amino acid composition.

Amino acid composition (%)	FM	SBM
Taurine	1.36 (± 0.07)	1.17 (± 0.01)
Hydroxyproline	0.90 (± 0.02)	0.21 (± 0.03)
Aspartic acid	4.78 (± 0.02)	5.24 (± 0.05)
Threonine	2.16 (± 0.02)	1.99 (± 0.01)
Serine	1.92 (± 0.01)	2.12 (± 0.05)
Glutamic acid	6.77 (± 0.05)	8.17 (± 0.09)
Proline	2.47 (± 0.02)	2.40 (± 0.07)
Glycine	4.00 (± 0.01)	2.32 (± 0.01)
Alanine	3.39 (± 0.01)	2.26 (± 0.01)
Cysteine	0.44 (± 0.01)	0.60 (± 0.00)
Valine	2.63 (± 0.01)	2.49 (± 0.01)
Methionine	1.44 (± 0.01)	1.23 (± 0.01)
Isoleucine	2.35 (± 0.01)	2.43 (± 0.01)
Leucine	3.76 (± 0.00)	3.77 (± 0.01)
Tyrosine	1.68 (± 0.02)	1.82 (± 0.00)
Phenylalanine	2.18 (± 0.00)	2.47 (± 0.00)
Lysine	4.19 (± 0.01)	5.43 (± 0.02)
Histidine	1.21 (± 0.00)	1.25 (± 0.00)
Arginine	3.23 (± 0.01)	3.51 (± 0.01)
Tryptophan	0.55 (± 0.02)	0.65 (± 0.01)
IDAA	23.70 (± 0.01)	25.21 (± 0.02)
DAA	21.72 (± 0.07)	24.05 (± 0.09)
TFAA	45.42 (± 0.08)	49.26 (± 0.10)

Diets were analyzed in triplicates. The values presented are the mean (± standard deviation). Indispensable amino acids (IDAA) = Ile, Leu, Lys, Met, Phe, Thr, Trp, Val, Arg, and His. Dispensable amino acids (DAA) = Ala, Asp, Taurine, Glu, Hyp, Gly, Pro, Ser, Cys, and Tyr.

*TFAA* total free amino acids.

### Diet analysis

Proximate composition of diets included quantification of the following: crude protein, crude lipid, moisture, and ash. Briefly, samples were analyzed for ash by combustion (550 °C for 5 h) in a muffle furnace (Lindberg Blue M, MA); crude protein (N × 6.25) using a Leco nitrogen analyser (Model FP-628, Leco Corporation, St. Joseph, MO); and crude lipid was extracted with chloroform–methanol (2:1, v/v), as described by Folch et al.^28^. The amino acid profile of each feed was analyzed utilizing the Association of Official Analytical Chemists, International (AOAC) Official Method 999.13. All dietary samples were analyzed in triplicates.

### Experimental design

**Figure 1:**
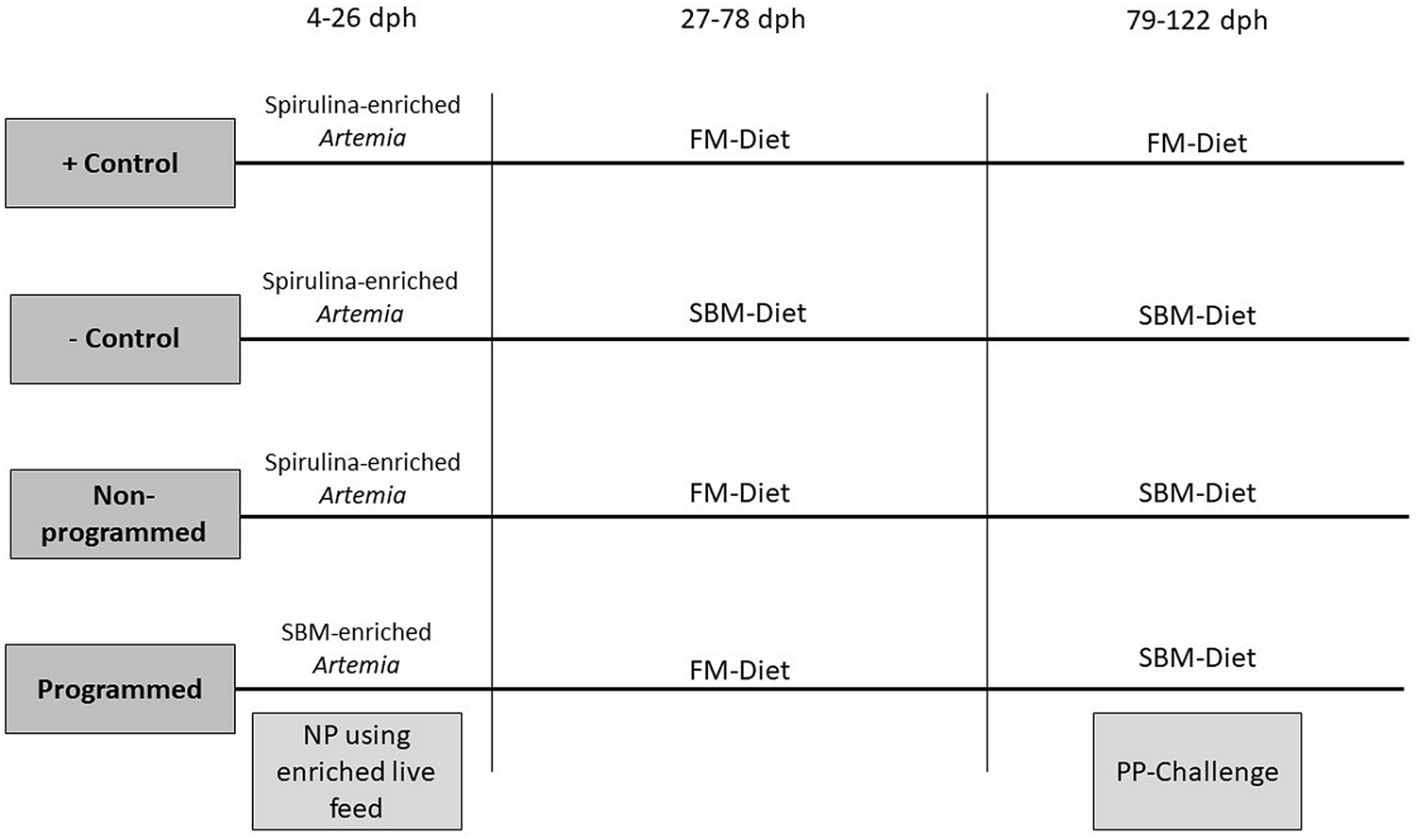
Feeding regime for each experimental group. *Dph* days post hatch, *FM* fishmeal, *PP* plant protein, *SBM* soybean meal, *NP*  nutritional programming.

At 4 days post hatch (dph), larval LMB (~ 0.0034 g) were randomly distributed into 12 (100 L) tanks with an initial average density of 867 (± 99) per tank. At 58 dph, the densities in each tank were reduced to 100 fish, and further reduced to 30 fish at 81 dph. The reduction in densities was done to ensure that the growth of the fish was not restricted by tank densities and that other measured parameters were not impacted by varying densities tanks. The experimental tanks started at a volume of 50 L and were increased to 100 L on 25 dph. There were 4 experimental groups utilized in this study, with 3 replicate tanks for each group. The first group was our programmed group. The programmed group was programmed with SBM through enriched *Artemia* nauplii (Brine Shrimp Direct, Ogden, UT) during 4–26 dph. After the programming, this group was fed with FM diet during 27–78 dph, and then exposed to the SBM diet during the “plant protein challenge” (PP-Challenge) (79–122 dph). The second group was our non-programmed group. This group underwent the same feeding regime as the programmed group, however it was not programmed with SBM during the live feed stage and, was fed spirulina enriched *Artemia* during 4–26 dph. The last two groups represent our control groups. The ( +) Control group and the (−) Control group both received regular spirulina enriched *Artemia* during 4–26 dph, and then were fed with the FM diet and SBM diet, respectively for the duration of the study (Fig. 1).

To ensure high feed intake, larval bass were fed to satiation up until 78 dph. Beginning at 79 dph, the fish were fed with a restricted feeding rate. The feeding rate was originally set by measuring the observed feed intake for each tank and setting the feeding level to the tank with the lowest feed intake. This ensured a consistent feeding rate across all tanks and ensured all food added to the tanks was consumed. Most importantly, this eliminated any potential bias that would have been caused by palatability or preference differences between the two diets that could have impacted the feed intake among groups. This helped ensure that any differences in growth performance would not be a function of varying feed intake levels. In addition, the feeding rate was adjusted daily, using an assumed FCR of 1, and also readjusted through observations of feed intake at each feeding. Also, a bi-weekly weighing was conducted during the restricted feeding period, in order to determine the actual biomass in each tank and, readjust the feeding rate accordingly. The feeding rates were 9% for 79–85 dph, 7% for 86–89 dph, 5% for 90–93 dph, 3% for 94–109 dph, and 2.5% for 110–122 dph.

### Live feed enrichment

**Figure 2:**
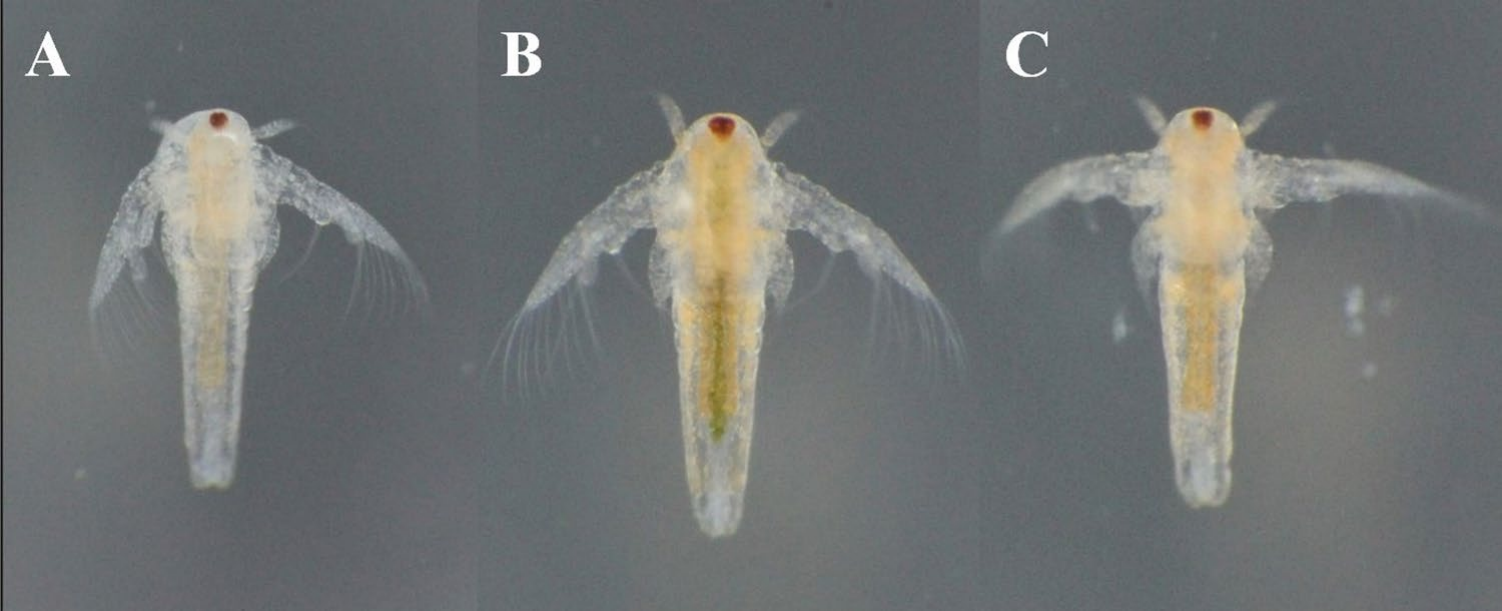
Enriched *Artemia* nauplii*.* Unenriched *Artemia* **(A)** are shown next to *Artemia* that were enriched for 3 h with spirulina **(B)** and SBM **(C)**.

Finely ground particles (< 0.25 mm) of SBM were mixed with deionized water to form a solution. The formulation for the solution was 1-part dry powder to 19 parts deionized water. The solution was thoroughly homogenized and filtered through 150 µm to ensure it was able to be absorbed (filtered) by the *Artemia* naupli. The *Artemia* were hatched and after 8 h (the time after which the nauplii start the filtering process^25^) were enriched for at least 3 h prior to being fed to the fish. Photographs of enriched *Artemia* are shown in Fig. 2. Water in the enrichment jars was changed twice a day to remove waste, and the enrichment solution was continuously added throughout the day to ensure a constant supply of enrichment solution in the water (Suppl. information).

### Sampling and measuring

At the conclusion of the study (122 dph), three fish from each tank were randomly sampled, euthanized with MS-222, and their intestines dissected and stored in a 10% formalin solution for histology analyses. In addition, 3 fish from each tank were euthanized with MS-222 and their intestines harvested and stored in RNALater (Sigma-Aldrich, St. Louis, MO, USA) for future analysis of gene expression. For free amino acid analysis, three fish were sampled from each tank at 3 and 24 h after feeding at the conclusion of the study and, stored at − 80 C until analysis.

Fish were weighed on a bi-weekly basis beginning at 79 dph in order to measure biomass and adjust feeding rates. The average weight per fish of each group was measured at the end of the study, as was the percent weight gain of each group during the study. Average weight was calculated for each tank by dividing the final biomass by the number of fish in the tank. Growth parameters in this study were calculated using the following formulas:

$${\text{Weight gain }}\left( \% \right) \, = \frac{{\text{Weight gain (g)}}}{{{\text{Initial weight }}\left( {\text{g}} \right) }} \times 100$$

$${\text{Feed Efficiency }}\left( {{\text{FE}}} \right) \, = {\text{ Weight Gain }}\left( {\text{g}} \right)/{\text{Feed Intake }}\left( {\text{g}} \right)$$

### Gene expression analysis

Intestinal samples were processed using TRIzol Reagent (Ambion, Foster City, CA, USA) and RNA was extracted and purified using the On-Column PureLink DNase Treatment (PureLink RNA Mini Kit and PureLink DNase, Invitrogen, Carlsbad, CA, USA) following the manufacturer’s instructions. Once purified, the ng/µl of each RNA sample was obtained using a spectrophotometer (Nanodrop 2000c, Thermo Fisher Scientific, Waltham, MA, USA). From this point, 2 µg of RNA from each sample was reverse transcribed using the High Capacity cDNA Reverse Transcription Kit (Applied Biosystems, Foster City, CA, USA) to obtain a 20 µl cDNA solution. The cDNA solutions were then added to a tube with 380 µl of water, to produce the cDNA sample for each tank. Gene expression of each cDNA sample was measured using a Bio-Rad (Hercules, CA, USA) CFX96 Real-Time PCR System. Each qPCR reaction mixture (20 µl) contained 9 µl of cDNA sample, 10 µl of PowerUp SYBR Green Master Mix (Thermo Fisher Scientific, Waltham, MA, USA), and 0.5 µl of 800 nM each of forward and reverse primers. Primers (Table 3) were synthesized by Integrated DNA Technologies (Coralville, IA, USA). Each qPCR reaction was run in technical duplicates. The qPCR cycle consisted of 95 °C for 10 min, followed by 40 cycles of 95 °C for 20 s and 60 °C for 35 s, followed by a melting curve to ensure the amplification of only a single product in each well. Relative gene expression was calculated using the 2−$\Delta \Delta$Ct method, normalizing the target gene expression to the expression of β*-actin* (reference gene)^29^. The expression of genes in in the programmed and non-programmed groups were then compared to that of the (-) Control group to observe how the expression varied between the three groups that received the PP-based diet.

**Table 3:** Primers used for gene expression analysis.

Gene	Sequence	Function	Source
*IL-1*	F: CAAGATGCCTAAGGGACTGGA	Proinflammatory cytokine	^28^
	R: AGGTGAACTTTGCGGTTCTC		
*IL-8*	F: GAGCCATTTTTCCTGGTGACT		
	R: TCCTCATTGGTGCTGAAAGATC		
*IL-12p40*	F: TCTTCCATCCTTGTGGTCTTCC		
	R: CAGTTCCAGGTCAAAGTGGTC		
*tnf-α*	F: CTAGTGAAGAACCAGATTGT		
	R: AGGAGACTCTGAACGATG		
*β-actin*	F: CCACCACAGCCGAGAGGGAA	Reference gene	
	R: TCATGGTGGATGGGGCCAGG		

### Histological analysis

The histological analysis followed the method described in Molinari et al.^26^. Histological slides were prepared by Saffron Scientific Histology Services (Carbondale, IL). Intestines previously fixed in 10% neutral buffered formalin were processed to paraffin using a Sakura enclosed automated tissue processor (Netherlands). The three representative areas of LMB intestines were orientated for cross sections embedded together in the same block. Five-micrometer serial sections were cut with a Leica manual microtome (Buffalo Grover, IL) and placed on water bath at 44 °C. Sections were placed on positive charged slides. After drying, slides were stained with hematoxylin and eosin and cover-slipped using acrylic mounting media. The histological analysis focused on the hind-gut portions of fish digestive tract. Pictures of each slide at 50X magnification were obtained using a microscope (Leica DMI 300B) and camera (Leica DMC 290) combination, with the software LAS V4.4 (Leica Camera, Wetzler, Germany, https://www.leica-microsystems.com). Pictures were taken of 3 samples from each tank, with 9 pictures being analyzed for each group. From these pictures, individual lengths and widths were taken of intact villi using ImageJ (NIH, Betheseda, MD, USA). Length and width data were measured in the distal portion of the intestine. Villus length was measured from the tip of the villus to the luminal surface, and villi width was measured across the base of the villus at the luminal surface. The length-to-width ratio of each villus was determined by dividing the length by the width.

### Free amino acid analysis

Muscle samples obtained from the frozen fish (− 80 ºC) were used for the analysis. Those muscle samples of three fish from each tank were combined and homogenized together with 0.1 M HCl in 1:9 (w/v) and spun at 12,000*g* (4 ºC, 15 min). Supernatants were collected, filtered (Milipore, 10 kDa cutoff at 15,000*g*, 4 ºC, 30 min) and later diluted with 0.1 M HCl (1:19 v/v) containing norvaline and sarcosine (40 µM) as internal standards. Blanks (0.1 M HCl + 40 μM norvaline and sarcosine) and external standards (Sigma acid/neutral and basic amino acids) were prepared along with the sample preparation. The same concentration of glutamine in 0.1 M HCl as an external standard was prepared and added to the basic amino acids standard. Free amino acids were quantified using Shimadzu Prominence Nexera—i LC-2040C Plus (Shimadzu, Japan) according to the Shimadzu protocol No. L529 with modifications^30^. Free amino acid concentrations (expressed as µmol/kg wet body weight) were calculated in LabSolutions software version 5.92 (Shimadzu, Japan, https://shimadzu.com.au/labsolutions) using internal and external standards.

### Statistical analysis

Results are presented as means (± standard deviation). One-way ANOVA was used to test the data, and a Tukey test was run to test the differences between groups. Due to the high variability of FAA concentrations, an LSD test was run to detect differences between groups. Differences between groups are considered significant at *p* values < 0.05. Statistical analysis was run using R version 3.5.2 (Boston, MA).

## Results

### Growth performance

At the conclusion of the study (at the end of the PP Challenge), the (−) Control group had a significantly lower average weight compared to the other three groups (*p* = 0.0001) (Table 4). The (%) weight gains presented in the tables were calculated to show the weight gain of each group from the start (79 dph) to the end of the PP-Challenge (122 dph). The programmed group had a significantly higher weight gain than the non-programmed group (*p* = 0.005). The programmed group’s (%) weight gain was also not significantly different from the ( +) Control group, which was still being fed FM-diet during the PP-Challenge. In addition, the programmed group had a significantly higher FE (*p* = 0.033) compared to the non-programmed group. The (−) Control group did have the highest (%) weight gain (593.22 ± 27.96) during the challenge, due to poor performance and significantly smaller average weight than the other groups at the start of the challenge caused by continued SBM diet feeding throughout the study.

**Table 4:** Treatment effect on growth performance measures.

Group	Initial weight (g)	Final weight (g)	Weight gain (g)	Weight gain (%)	FE
+ Control	5.76^b^ (± 0.87)	27.04^b^ (± 2.76)	21.28^c^ (± 1.92)	372.05^b^ (± 27.04)	0.69^b^ (± 0.02)
− Control	2.18^a^ (± 0.11)	15.19^a^ (± 1.38)	13.00^a^ (± 1.27)	593.22^c^ (± 27.96)	0.96^c^ (± 0.03)
Non-programmed	6.36^b^ (± 0.66)	23.91^b^ (± 0.91)	17.55^b^ (± 0.27)	277.79^a^ (± 26.50)	0.59^a^ (± 0.04)
Programmed	5.45^b^ (± 0.21)	24.89^b^ (± 1.04)	19.44^bc^ (± 0.96)	357.25^b^ (± 18.84)	0.68^b^ (± 0.03)

Average weight represents the final weight of the fish at the conclusion of the study. Weight gain represents the weight gain during the PP-Challenge (79–122 dph). Values are presented as means (± std. dev). Superscript letters indicate statistical significance between groups. The significance was determined using a One-Way ANOVA and a Tukey test with a p value < 0.05.

No significant differences were observed in survival between groups during the larval programming stage (4–26 dph), with an overall survival of 90.29 (± 2.64). During the middle stage of the study (27–28 dph), after the programming stage and before the PP-Challenge, the (−) Control group (47.19 ± 4.97) had a significantly lower survival than both the ( +) Control group (69.87 ± 7.50, *p* = 0.0194) and the programmed group (79.10 ± 7.79, *p* = 0.0026). The survival of the non-programmed group (65.26 ± 7.88) during this stage was not significantly different from any of the other groups. No significant differences in survival were observed during the PP-Challenge, with only the (−) Control group (92.22 ± 6.93) exhibiting any more than 1 mortality per tank throughout the whole period.

### Intestinal health

**Figure 3:**
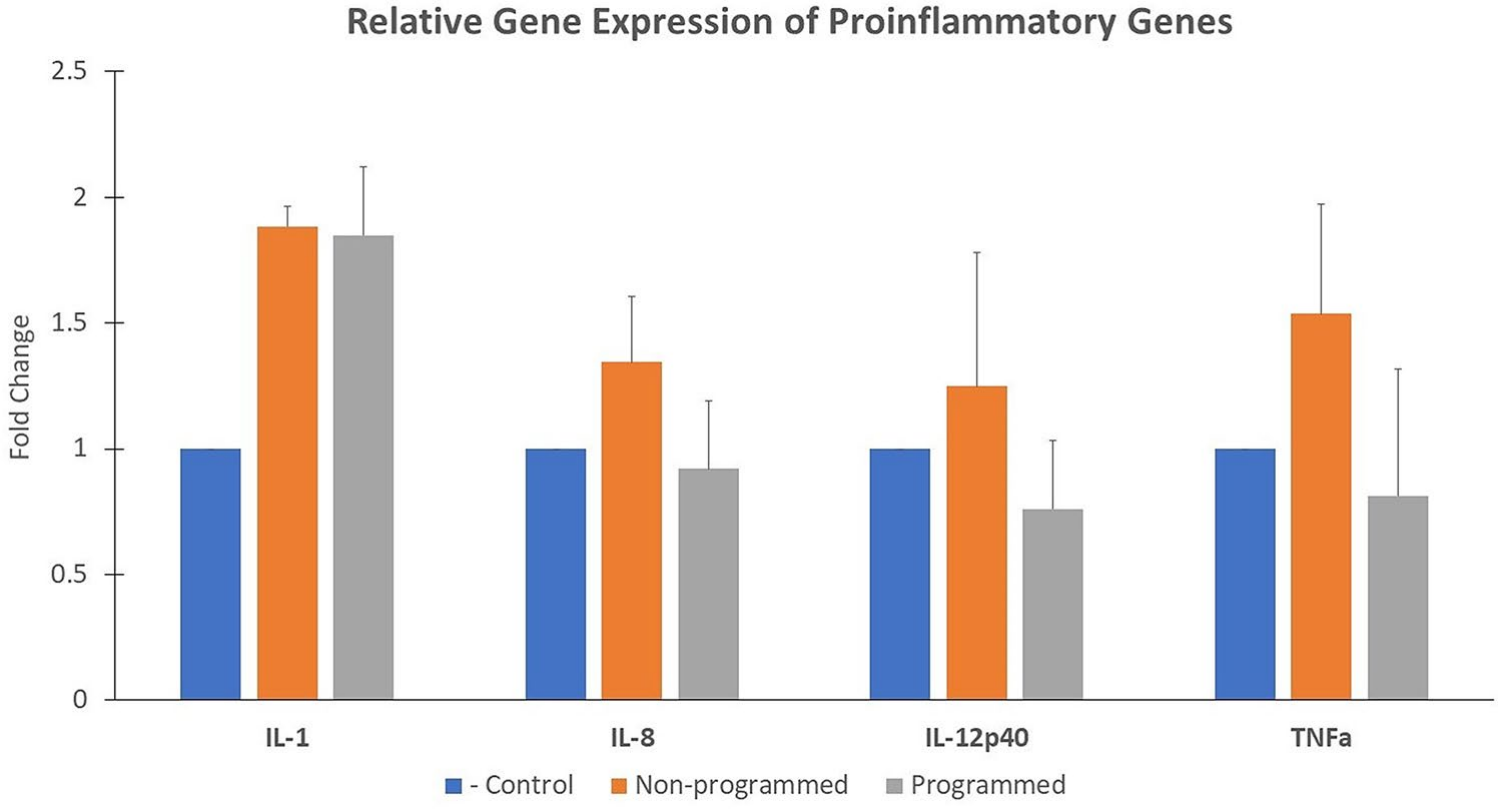
Relative gene expression. Relative gene expression of the group is represented as a fold change relative to the negative control group (fold change = 1). Values provided are mean fold change + S.E.M (standard error of the mean). Results of One-way ANOVA and Tukey test are shown on graphs when significant. No significant differences were detected.

There were no significant differences in the expression of the pro-inflammatory cytokines across the different groups (Fig. 3).

**Figure 4:**
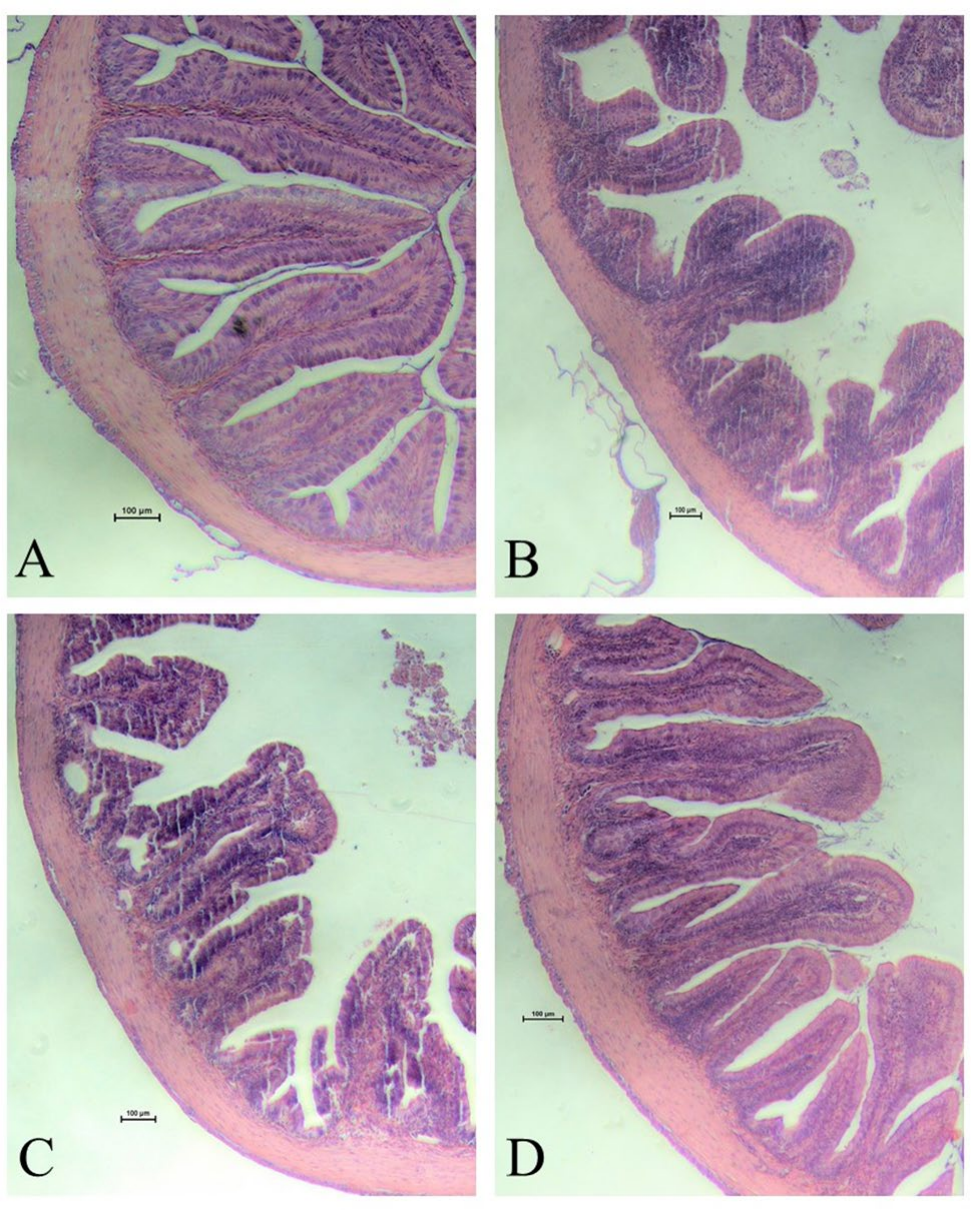
Cross-section of distal intestine samples. Histological samples of distal intestines from largemouth bass. **(A)** ( +) Control; **(B)** (−) control; **(C)** non-programmed; **(D)** programmed.

Looking at the histology data (Table 5), the ( +) Control and programmed groups both had significantly longer distal villi than the (−) Control and non-programmed groups (*p* = 0.0001). In addition, the ( +) Control had significantly wider villi than the (−) Control (*p* = 0.005), but the programmed and non-programmed groups were not significantly different from either group. Using the length and width values, the length-to-width ratio of the villi was calculated to get a representation of its surface area. The ( +) Control group had a significantly higher length-to-width ratio than the non-programmed group (*p* = 0.002). The (−) Control group showed a numerically lower length-to-width ratio, although it was not significantly different from any of the other groups. Between the two non-control groups, the programmed group had a significantly higher length-to-width ratio of their distal villi, compared to the non-programmed group (*p* = 0.0001). Figure 4 presents examples of histology samples analyzed from each group. The raw data from the intestinal health analyses are provided in Supplementary Information 1.

**Table 5:** Treatment effect on the villus measurements in the distal portion of the intestine. Values are presented as means (± std. dev).

Group	Distal villus measurements		
	Length (µm)	Width (µm)	Length to width ratio
+ Control	517.54^b^ (± 81.42)	128.93^b^ (± 36.00)	4.27^b^ (± 1.20)
− Control	382.90^a^ (± 76.62)	106.28^a^ (± 31.76)	3.80^ab^ (± 1.05)
Non-programmed	400.25^a^ (± 121.40)	126.80^ab^ (± 47.54)	3.37^a^ (± 1.13)
Programmed	477.46^b^ (± 60.51)	117.46^ab^ (± 28.45)	4.29^b^ (± 1.13)

Superscript letters indicate statistical significance between groups. The significance was determined using a One-Way ANOVA and a Tukey test with a p value < 0.05.

### Free amino acid composition

The results for muscle FAA postprandial levels 3-h after feeding are presented in Table 6. All data from the FAA analysis is provided in Supplementary Information 2. Among the 10 indispensable amino acids (IDAA) analyzed, half showed significant differences between groups. The concentration of isoleucine was significantly higher in the (−) Control (*p* = 0.046) and FM-PP (*p* = 0.022) groups, compared to the ( +) Control. The programmed group had a significantly higher concentration of leucine than the ( +) Control group (*p* = 0.036). The ( +) Control group had a significantly lower level of both lysine (*p* = 0.0001) and histidine (*p* = 0.0008) than all of the other groups. The level of histidine was also significantly higher in the (−) Control group than in the non-programmed group (*p* = 0.037). The (−) Control group also had a significantly higher level of arginine, compared to the other groups (*p* = 0.036).

**Table 6:** Dietary treatment effect on the muscle concentration of free amino acids.

Amino acid (µmol/g)	+ Control	− Control	Non-programmed	Programmed
Aspartic acid	0.04^a^ (± 0.00)	0.09^b^ (± 0.02)	0.06^ab^ (± 0.00)	0.05^a^ (± 0.01)
Glutamic acid	0.45 (± 0.03)	0.51 (± 0.10)	0.45 (± 0.03)	0.45 (± 0.03)
Asparagine	0.24^a^ (± 0.02)	1.22^c^ (± 0.22)	0.81^b^ (± 0.16)	0.92^b^ (± 0.15)
Serine	0.28^b^ (± 0.04)	0.19^a^ (± 0.04)	0.21^a^ (± 0.03)	0.18^a^ (± 0.04)
Glutamine	0.49^a^ (± 0.08)	0.82^b^ (± 0.13)	0.60^ab^ (± 0.10)	0.65^ab^ (± 0.15)
Histidine	2.44^a^ (± 0.25)	4.08^c^ (± 0.37)	3.46^b^ (± 0.28)	3.80^bc^ (± 0.32)
Glycine	4.02^b^ (± 1.41)	1.90^b^ (± 0.34)	2.75^ab^ (± 0.55)	2.25^a^ (± 0.35)
Threonine	0.56 (± 0.48)	0.59 (± 0.07)	0.52 (± 0.06)	0.52 (± 0.03)
Arginine	0.10^a^ (± 0.02)	0.14^b^ (± 0.01)	0.11^a^ (± 0.01)	0.12^a^ (± 0.01)
Alanine	0.47^a^ (± 0.02)	0.89^c^ (± 0.07)	0.68^b^ (± 0.09)	0.79^bc^ (± 0.05)
Tyrosine	0.09^b^ (± 0.02)	0.07^a^ (± 0.01)	0.08^ab^ (± 0.00)	0.07^ab^ (± 0.01)
Methionine	3.28 (± 0.43)	3.46 (± 0.15)	3.15 (± 0.18)	3.37 (± 0.07)
Valine	0.60 (± 0.06)	0.65 (± 0.05)	0.58 (± 0.05)	0.64 (± 0.03)
Tryptophan	0.01 (± 0.00)	0.01 (± 0.00)	0.01 (± 0.00)	0.01 (± 0.00)
Phenylalanine	0.05 (± 0.01)	0.04 (± 0.01)	0.04 (± 0.00)	0.04 (± 0.00)
Isoleucine	0.04^a^ (± 0.00)	0.05^b^ (± 0.02)	0.06^b^ (± 0.01)	0.05^ab^ (± 0.00)
Leucine	0.00^a^ (± 0.00)	0.05^ab^ (± 0.08)	0.08^ab^ (± 0.07)	0.11^b^ (± 0.00)
Proline	0.27^a^ (± 0.03)	0.48^bc^ (± 0.09)	0.30^ab^ (± 0.09)	0.52^c^ (± 0.15)
Lysine	0.15^a^ (± 0.04)	1.09^b^ (± 0.17)	0.95^b^ (± 0.19)	1.06^b^ (± 0.05)
IDAA	7.23^a^ (± 0.26)	10.17^c^ (± 0.75)	8.96^b^ (± 0.12)	9.70^bc^ (± 0.44)
DAA	6.34 (± 1.52)	6.16 (± 0.63)	5.93 (± 0.50)	5.89 (± 0.04)
TFAA	13.57^a^ (± 1.28)	16.33^b^ (± 1.28)	14.89^ab^ (± 0.60)	15.60^b^ (± 0.46)

The values presented are post-prandial levels, 3 h after feeding. Values are presented as means (± std. dev). Superscript letters indicate statistical significance between groups. The significance was determined using a One-way ANOVA and an LSD test with a p value < 0.05. Indispensable amino acids (IDAA) = Ile, Leu, Lys, Met, Phe, Thr, Trp, Val, Arg, and His. Dispensable amino acids (DAA) = Ala, Asp, Asn, Glu, Gln, Gly, Pro, Ser, and Tyr.

*TFAA*  total free amino acids.

Among the 9 dispensable amino acids (DAA) analyzed, 8 showed significant differences among groups. The level of aspartic acid was significantly higher in the (−) Control group, compared to the ( +) Control (*p* = 0.002) and programmed group (*p* = 0.039). Asparagine had the highest concentration in the (−) Control group and the lowest in the ( +) Control group, with both groups significantly different from the non-programmed and programmed groups (*p* = 0.0004). The same results were observed in the alanine levels, except that the programmed group was not significantly different from the (−) Control group. The ( +) Control group had a significantly higher concentration of tyrosine (*p* = 0.022), and a lower concentration of glutamine (*p* = 0.009), compared to the (−) Control group. The glycine concentration was significantly lower in the programmed group, compared to the ( +) and (−) Control groups (*p* = 0.011, *p* = 0.026). The ( +) Control group had a significantly higher level of serine, compared to all other groups (*p* = 0.039).

A main focus of these results was to look at differences between the programmed and non-programmed groups. There were no significant differences between these two groups for any FAA tested, except for proline, which was significantly higher in the programmed group (*p* = 0.023). The concentration of IDAA was significantly higher in all groups compared to the ( +) Control (*p* = 0.0002). In addition, the (−) Control group had a significantly higher IDAA concentration than the non-programmed group (*p* = 0.012). There was no significance in the concentration of dispensable amino acids (DAA) among groups, but for total free amino acids (TFAA), the (−) Control (*p* = 0.008) and programmed (*p* = 0.034) groups had significantly higher levels than the ( +) Control group.

## Discussion

Previous studies have found that NP using dry feed improves the utilization of dietary PP^3,13–15^. Kwasek et al.^13^ also tested the use of live feed for NP and found that, although non-significant, the live feed-programmed fish achieved numerically higher weight gain on a PP-based diet than non-programmed fish. The authors argued, however, that prolonged feeding duration could have made the result significant but the model choice (zebrafish *Danio rerio*) and its sexual dimorphism forced investigators to terminate the study earlier. This present experiment focuses on aquaculture species, LMB, and found that NP through live feed enrichment significantly improved the growth performance of LMB fed an SBM-diet during pre-adult stages. The programmed fish achieved an average (%) weight gain of 357% compared to a (%) weight gain of 278% for the non-programmed group. More significantly, the programmed group had close to the same (%) weight gain and FE feeding on a PP-diet as the ( +) Control group which was fed a diet based 100% on marine animal protein sources. The high weight gain observed in the (−) Control group is misleading due to its significantly lower initial weight at the start of the PP-Challenge. The average initial weight of the (−) Control group (2.18 g (± 0.11)) was more than 50% smaller than any of the other groups at that stage. Over the 43-day span of the PP-Challenge the (−) Control had a (%) weight gain of 593.22 (± 27.96). When the other three groups were of a comparable initial size (~ 2 g), their (%) weight gain over a shorter period (37-day span; 58–95 dph) similarly amounted 545.68 (± 96.17) compared to the lower (%) weight gain of 335.70 (± 48.73) achieved during PP-Challenge when the average initial fish size was 5.86 g (± 0.69). Thus, based on the growth trends for the fish in the other groups in this study, it can be assumed that the increased (%) weight gain in the (−) Control group can be attributed to its smaller size (and hence higher growth rate) at the start of the PP-Challenge. In addition, because of the low survival of the (−) Control group before PP Challenge (~ 47%), the growth response presented by this group fed SBM diet during the last 43 days of the study was likely a result of genetic selection of individuals characterized by higher resistance to the negative effects of SBM.

Other carnivorous species have been found to be negatively impacted by the inclusion of SBM into their diets. In spotted rose snapper (*Lutjanus guttatus*), a FM-replacement level of 60% with SBM significantly reduced the growth rate of the fish^8^. The same effects were observed at an even lower inclusion level of 30% SBM in Korean rockfish (*Sebastes schlegeli*)^10^. Similar to the other carnivorous species, LMB are also known to be sensitive to high SBM inclusion levels in their diet. A previous study found that the inclusion of dietary SBM for LMB led to a significant decrease in both growth and digestibility, even at a level of 50% FM replacement^31^. This study provides support that, with the use of NP with SBM through live feed, a carnivorous aquaculture species like LMB can grow as efficiently on a ~ 60% soy-based diet, as they do on a typical FM-based diet.

The mechanism behind NP is believed to occur through epigenetic modifications in the DNA. In a review by Lillycrop and Burdge^32^, it was determined that nutritional exposure in early life stages alters the epigenome and consequently, affects the regulation of certain genes in later life stages. This possible epigenetic effect of early exposure to PP has been observed to affect the expression of genes associated with digestive hormones^13^ and also appetite regulation^33^. Kwasek et al.^13^ found that NP in zebrafish had a significant impact on *ghrelin, cholecystokinin,* and *neuropeptide Y* expression. Additionally, NP has been observed to impact the expression of intestinal peptide transporter *PepT1*^26^. The same epigenetic effect that NP has had on appetite and digestion related genes, has also been observed in intestinal inflammation-related genes. In this study, we looked at the expression of genes associated with intestinal inflammation in LMB and their response to re-exposure to SBM in the later life stages, similar to Perera and Yufera^34^. More specifically, this study analyzed the expression of proinflammatory cytokines *IL-1* (*Interleukin-1*), *IL-8* (*Interleukin-8*), *tnf- α* (*tumor necrosis factor alpha*), and *IL-12p40* (*Interleukin 12 subunit p40*). Hedrera et al.^35^ found that both *IL-1* and *IL-8* were significantly up-regulated in the intestines of zebrafish that were exposed to SBM. In addition, the up-regulation of *tnf-α* has been found to be a result of SBM induced intestinal inflammation^36^. Wang et al.^37^ observed that the genes *IL-1, tnf- α,* and *IL-12p40* all showed an increased expression with increases in SBM levels in the diet. This finding is consistent with another programming study that found that NP of zebrafish significantly downregulated expression of pro-inflammatory cytokines when the fish were reintroduced to SBM later in life^34^. The findings of these studies may signify that the intestines of the programmed fish have adapted to the SBM leading to reduced level of inflammation induced by the SBM diets. This study however, did not find a significant effect of NP on the intestinal inflammatory cytokines in LMB.

SBM inclusion in diets has been found to trigger an inflammatory response that negatively affects the morphology of the intestines of fish. The SBM induced inflammation (enteritis) is characterized by shorter villi and a decreased capacity for absorption in the epithelial enterocytes^6,38–41^. Zhang et al.^40^ found that 75% replacement of FM with SBM resulted in significantly shorter intestinal villi and, correlated with increased enteritis and a significantly reduced growth rate in Japanese sea bass (*Lateolabrax japonicus*). Similar results were observed in beluga sturgeon (*Huso huso*), where SBM inclusion resulted in shorter and wider distal villi^41^. In addition, Wang et al.^6^ observed that the complete replacement of FM with SBM in diets reduced the length of the intestinal villi and also the surface area of the villi. The negative impact on the intestinal morphology led to a significantly reduced growth rate and feeding efficiency for the group fed a 100% SBM-based diet^6^. Previously, NP has been found to increase the surface area for absorption in the intestine, represented by longer villi and a higher length-to-width ratio of the villi^13^. This study found that the programmed group had longer distal villi and also a higher length-to-width ratio of the distal villi, compared to the non-programmed group. It is possible that the programming may have made the intestines of the bass more resistant to the inflammatory properties in SBM, leading to increased surface area for absorption in the intestine and ultimately improved nutrient absorption capacity reflected by higher weight gain during the PP-Challenge.

The muscle FAA results in this study reveal a significant difference between the utilization of dietary FM and SBM. Previous studies have found that FM replacement with PP leads to a significant delay in the peak of FAA in the body, which means that the timing of post-prandial FAA peak in fish tissues is significantly affected by the dietary protein source^42^. Gomez-Requeni et al.^43^, observed that the post-prandial level of FAA in the muscle of gilthead sea bream (*Sparus aurata*) was significantly higher in fish that were fed with a PP-based diet, compared to a FM-based diet. The authors determined that this increase in muscle TFAA signified a lower utilization rate of the dietary protein and, was correlated with a reduced growth rate in the PP-fed group^43^. The results of this study might support these findings. The levels of IDAA and TFAA were significantly higher in the PP-fed (−) Control group, compared to the FM-fed ( +) Control group. The same trend can be observed in many of the individual free amino acids like asparagine, histidine, alanine, and lysine, which were all significantly lower in the ( +) Control group compared to the three PP-fed groups. Similar to the study done by Gomez-Requeni et al.^43^ the high TFAA and IDAA levels in the (−) Control group were also associated with a significant reduction in growth rate for the PP-fed fish. We can assume that the significant differences among FAA concentrations in the study between the ( +) Control group and the three PP-fed groups are attributed to the different dietary protein source. The effect of the difference in digestive efficiency between the two dietary protein sources could be further amplified by the fact that LMB is a warm-water, carnivorous species. One study done on rainbow trout, a cold-water, carnivorous species, found that the peak FAA levels in the plasma occurred ~ 12 h after feeding with FM and ~ 21 h after feeding with SBM^44^. Another study done on rainbow trout observed a similar delay, with FAA peaking in the plasma at 6–8 h after feeding with FM and 12 h after feeding with PP^42^. While those studies were done on cold-water fish, it has been found that there is a temperature effect on amino acid absorption, with absorption rates increasing with rising temperatures^45^. In a study done on yellowtail (*Seriola quinqueradiata*), a warm-water carnivore, the peaks of FAA appeared just 2–4 h after feeding with raw fish^46^. Based on these studies, it is apparent that in carnivorous species, amino acids are absorbed much more rapidly from FM-based diets compared to PP-based diets, and that amino acid absorption and utilization is quicker in warm-water species, compared to cold-water species. Thus, it is feasible that at 3 h after feeding, more of the FAA in the ( +) Control group may have been absorbed and utilized prior to sampling, while the delayed digestion of the SBM-diet in the other groups may have caused the FAA to just reach the muscle pool at the time of sampling.

However, the results of the FAA analysis for this study showed minimal difference between the programmed and non-programmed groups. The lack of differences between the two groups may be linked to the relatively low dynasticity of the muscle FAA pool. Mente et al.^47^ found that the white muscle FAA concentrations of Atlantic salmon (*Salmo salar*) remained undifferentiated for at least 12 h after being fed with diets containing varying levels of maize gluten as a protein source. The same study determined that the white muscle FAA pool may be resistant to significant post-prandial changes in FAA concentrations immediately after feeding^47^, whereas FAA pools in other areas of the fish body, including blood plasma, have been shown to be more responsive to FAA changes after feeding^48^. Among the muscle FAA measured, the only significant difference between the programmed and non-programmed groups was the concentration of free proline. Proline is a DAA that plays a key role in protein synthesis, including the synthesis of collagen^49^. l-proline as a dietary supplement has been found to increase growth performance in pigs and, improve the functionality of the gastrointestinal tract^50^. While it is unlikely that the higher concentration of proline had a significant effect on the increased growth performance of the programmed fish, it is still worth noting due to its functional role in tissue protein accretion.

## Conclusion

The results from this study support the use of live feed as a vehicle of SBM for NP in larval LMB. The programmed bass experienced significantly higher weight gain (%) during the PP-Challenge, compared to the non-programmed fish and achieved a weight gain similar to that of the bass that were fed FM. The mechanism behind the programming seemed to lie in the intestine. While no significant effect was observed on the expression on inflammatory cytokines, the significantly higher length-to-width ratio of the distal villi signifies an increased surface area for nutrient absorption in the intestine as a result of the NP. The programming did not have a significant effect on FAA concentrations, except that free proline had a higher concentration in the programmed group.

By using enriched live feed to program at first feeding, we are able to begin introduction of PP to the larval fish at the earliest possible point (first feeding) and, prevent the programming process from having a negative effect on the fish development in its early life stages. Further research would be beneficial into determining the viability of live feed NP across a wider variety of aquaculture species, and also providing a deeper look into the physiological mechanisms behind NP. The findings from this study are important for the industry of aquaculture allowing for NP at first feeding, without disrupting the natural feeding regime of the fish ultimately leading to increased utilization of feeds containing SBM and hence reduction in costs of commercial PP-based dietary formulations.

## Supplementary Information

Supplementary Information 1.

Supplementary Information 2.

## Data Availability

All data is available in the supplementary information files.
